# Epidemiological Characteristics and Incidence Trends of Hemorrhagic Fever With Renal Syndrome in Shandong Province, China: Retrospective Observational Study With Forecasting

**DOI:** 10.2196/99524

**Published:** 2026-10-16

**Authors:** Qing Duan, Ti Liu, Yinlong Li, Suying Guo, Shiyi Huang, Jing Xu, Ruixiao Li, Zengqiang Kou

**Affiliations:** 1 National Institute of Parasitic Diseases, Chinese Center for Disease Control and Prevention; Chinese Center for Tropical Diseases Research; NHC Key Laboratory of Parasite and Vector Biology; WHO Collaborating Centre for Tropical Diseases National Center for International Research on Tropical Diseases, Ministry of Science and Technology Shanghai China; 2 Shandong Provincial Center for Disease Control and Prevention Jinan China; 3 Shandong Provincial Key Laboratory of Intelligent Monitoring, Early Warning, Prevention and Control for Infectious Diseases Jinan China; 4 School of Global Health, Chinese Center for Tropical Diseases Research, Shanghai Jiao Tong University School of Medicine Shanghai China

**Keywords:** hemorrhagic fever with renal syndrome, seasonal autoregressive integrated moving average, SARIMA, Prophet, Holt-Winters, Extreme Gradient Boosting, XGBoost, prediction

## Abstract

**Background:**

Hemorrhagic fever with renal syndrome (HFRS) is a severe natural focal infectious disease that remains a major public health concern in Shandong Province, China. Understanding the epidemiological characteristics of HFRS and forecasting its incidence are crucial to provide guidance for policymaking and implement timely interventions against this disease.

**Objective:**

This study examined the long-term epidemiological characteristics of HFRS based on 15 years of surveillance data; the analysis used 4 time series models to predict its incidence trends. The aim was to provide a scientific basis for local disease control departments, thereby supporting more accurate prediction and effective prevention and control of HFRS in this region.

**Methods:**

HFRS case surveillance data in Shandong Province from 2010 to 2024 were collected through the China Information System for Disease Control and Prevention. Descriptive epidemiological methods were used to analyze the basic characteristics of HFRS. Four forecasting models, namely, seasonal autoregressive integrated moving average, Prophet, the Holt-Winters exponential smoothing method, and Extreme Gradient Boosting, were implemented in R to fit the time series and generate predictions. Their predictive performance was evaluated and compared using the root mean squared error, mean absolute error, and mean absolute percentage error.

**Results:**

From 2010 to 2024, a total of 15,184 HFRS cases were reported in Shandong Province, with 188 deaths. The annual incidence rate ranged from 0.45 to 1.84 per 100,000 population, showing an overall fluctuating downward trend. The peak incidence occurred during the autumn and winter months from October to December each year. Spatially, the eastern and southeastern regions of Shandong Province were the primary high-incidence areas. Demographically, the high-risk populations were mainly middle-aged and older adults, male individuals, and farmers. In the 2024 test set, the Holt-Winters model yielded the lowest prediction error metrics among the 4 models, with root mean squared error, mean absolute error, and mean absolute percentage error values of 7.56, 6.43, and 34.56%, respectively.

**Conclusions:**

The Holt-Winters model showed the lowest prediction error metrics among the 4 models, suggesting its potential utility for forecasting HFRS incidence. It is recommended to apply this model to enhance precise monitoring and implement targeted prevention and control measures focusing on key areas, peak periods, and high-risk populations.

## Introduction

Hemorrhagic fever with renal syndrome (HFRS) is a zoonotic disease, with rodents serving as the primary source of infection [1,2]. Humans are commonly infected through contact with rodent excreta or the resulting aerosols; currently, there is no conclusive evidence of human-to-human transmission of HFRS [3,4]. The disease is primarily characterized by acute fever, hemorrhage, and renal dysfunction, which can lead to a substantial disease burden [5]. HFRS first emerged during World War I and is now distributed across more than 70 countries and regions worldwide, although it is predominantly endemic to Eurasia [6]. China experiences the most severe HFRS epidemics worldwide, with cases having been reported in all 31 provinces (including autonomous regions and municipalities) [7]. The transmission of HFRS is jointly influenced by ecological, meteorological, and socioeconomic factors [8]. Meteorological factors such as temperature, precipitation, and humidity can directly affect rodent population dynamics as well as the survival and transmission of the virus in the environment [9]. Meanwhile, socioeconomic factors, including land use, urbanization, and human behaviors, also impact the risk of human exposure [10]. HFRS is an acute infectious disease caused by various genotypes of hantaviruses [11]. HFRS endemic areas in China are primarily classified into 3 categories, based on virus types and host animal species: the wild rodent–type endemic area, where HFRS is caused by the Hantaan virus (HTNV) and primarily involves dominant wild rodent species such as the striped field mouse; the domestic rodent–type endemic area, where HFRS is caused by the Seoul virus (SEOV) and primarily involves dominant domestic rodent species such as the brown rat; and the mixed-type endemic area, where both types coexist [12].

Shandong Province is one of the primary endemic areas for HFRS in China. Since the identification of the first case in 1968, cases have been reported annually [13-15]. Despite long-term efforts in surveillance, prevention, and control, HFRS in Shandong Province continues to exhibit complex temporal fluctuations, including seasonal peaks and multiyear cyclical variations [16]. Recent studies also suggest that the prevalence of HFRS in Shandong Province is characterized by distinct spatial heterogeneity and environment-driven features, highlighting the need to further enhance surveillance and early warning capabilities [17]. In 2004, China established a unified national web-based direct reporting system for infectious diseases, enabling real-time epidemic monitoring through a hierarchical reporting structure, which has laid a solid foundation for the forecasting and early warning of infectious diseases. Reliable forecasting results help optimize the allocation of health care resources, facilitate the timely issuance of health advisories, and enable the strengthening of vector control prior to the onset of anticipated epidemic peaks [18,19]. Time series modeling has emerged as a crucial method for predicting infectious disease epidemics [20]. Traditional models such as the seasonal autoregressive integrated moving average (SARIMA) model have been widely used to capture linear patterns and seasonal dependencies within HFRS incidence data [21]. Furthermore, exponential smoothing methods such as the Holt-Winters method offer considerable simplicity and practicality in handling trends and seasonality [22]. In recent years, the application of highly complex algorithmic models, including machine learning, has further enriched this research domain [23]. For instance, the Prophet model is well suited for time series with pronounced seasonal characteristics, whereas the Extreme Gradient Boosting (XGBoost) model demonstrates robust potential in forecasting structured epidemiological data [21,24]. However, in highly endemic areas for HFRS such as Shandong, research that systematically characterizes long-term epidemic patterns while simultaneously comparing the performance of classic and modern forecasting models remains relatively limited. Such studies are of profound significance for identifying forecasting tools that better meet the practical demands of public health.

Given the persistent prevalence of HFRS in Shandong Province and the lack of a systematic evaluation of time series forecasting models, this study conducted an in-depth analysis of the long-term epidemiological characteristics of HFRS based on 15 years of surveillance data and used 4 time series models to predict its incidence trends. The aim was to provide a scientific basis for local disease control departments, thereby supporting more accurate prediction and effective prevention and control of HFRS in this region.

## Methods

### Study Area

Located on the eastern coast of China, Shandong Province covers a total area of approximately 158,000 km^2^ and has a population exceeding 100 million, making it one of the country’s most populous and economically significant provinces (Figure 1). The province features a temperate monsoon climate with 4 distinct seasons. Its topography encompasses plains, hills, and coastal areas, providing diverse habitats for the primary host animals of HFRS. As a traditional high-incidence area for HFRS in China, Shandong has experienced long-term, continuous reporting of cases in both urban and rural areas. Its well-established public health surveillance system and significant disease burden make it an ideal region for studying the long-term epidemiological characteristics and incidence trends of HFRS.

**Figure 1:**
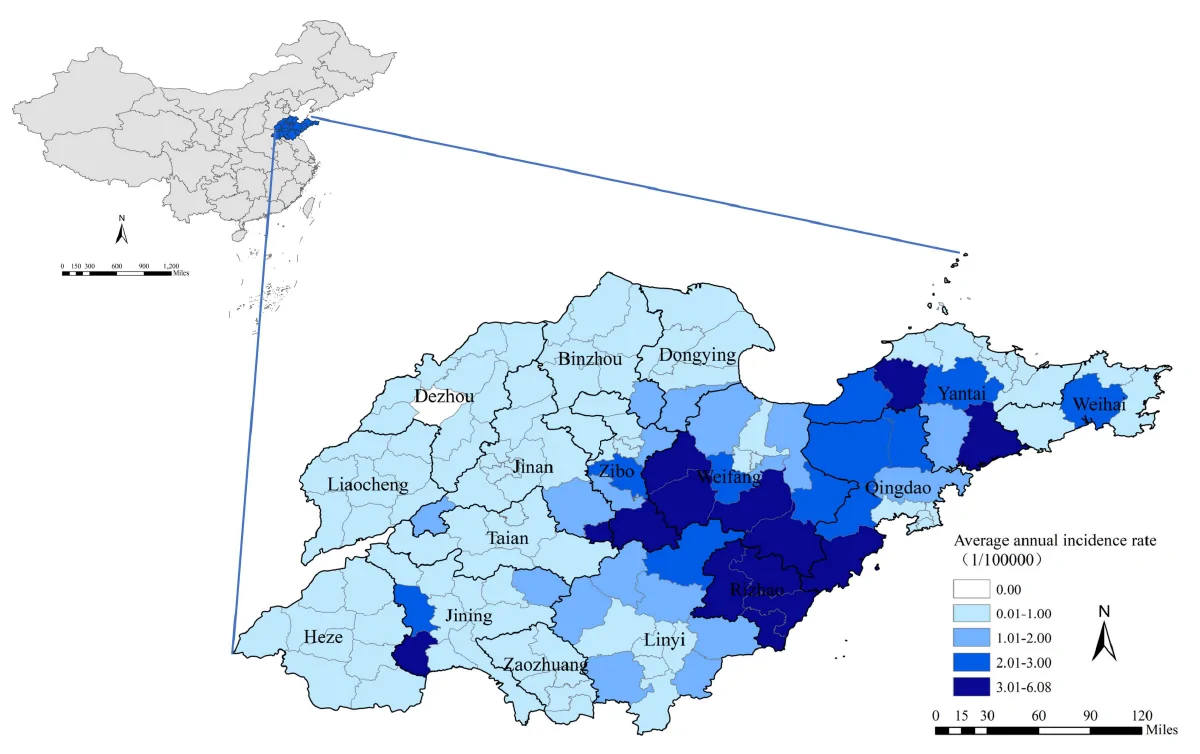
Geographical location of Shandong Province in China and the average annual incidence rate of hemorrhagic fever with renal syndrome from 2010 to 2024.

### Data Collection

HFRS case data were obtained from the China Information System for Disease Control and Prevention. This surveillance system collects case information based on the unified national diagnostic criteria for HFRS. Prior to analysis, all personally identifiable information was removed to protect patient privacy. According to the Law of the People’s Republic of China on the Prevention and Treatment of Infectious Diseases, HFRS is categorized as a class B infectious disease. In China, when patients with a class B infectious disease are identified by any level or type of medical institution, physicians are required to complete direct online reporting within 24 hours. This study collected HFRS case surveillance data in Shandong Province from January 2010 to December 2024, including variables such as sex, age, occupation, date of onset, and current residential address.

### Statistical Analysis

#### Overview

Descriptive epidemiological analyses were performed using the SPSS software (version 23.0; IBM Corp). Spatial mapping was conducted using the ArcGIS software (version 10.7; Esri). Additionally, packages including *forecast*, *prophet*, and *xgboost* in the R software (version 4.2.3; R Foundation for Statistical Computing) were used to construct 4 time series models (SARIMA, Prophet, Holt-Winters, and XGBoost), whereas the *ggplot2* package was used to visualize the results.

#### SARIMA Model

As an extension of the autoregressive integrated moving average model, the SARIMA model comprises both nonseasonal and seasonal components, with its general expression denoted as SARIMA(*p*, *d*, *q*) (*P*, *D*, *Q*)*s*. Specifically, *p*, *d*, and *q* represent the nonseasonal autoregressive order, differencing order, and moving average order, respectively; *P*, *D*, and *Q* denote the seasonal autoregressive order, seasonal differencing order, and seasonal moving average order, respectively; and *s* indicates the length of the seasonal period of the time series [25]. During model construction, the augmented Dickey-Fuller test was first used as a preliminary assessment of stationarity. A significance level of α=.05 was adopted, and a *P* value below .05 was considered indicative of stationarity. Seasonal-trend decomposition using locally estimated scatterplot smoothing (STL) was then applied to characterize the long-term trend and seasonal variation, and the seasonal period was determined accordingly. The autocorrelation function (ACF) and partial ACF were examined to characterize the temporal dependence structure. Candidate SARIMA models were subsequently screened using the *auto.arima* function, and the optimal model was selected according to the minimum corrected Akaike information criterion. Finally, residual autocorrelation was assessed using the Ljung-Box *Q* test at a significance level of α=.05 [26].

#### Prophet Model

The Prophet model is a time series forecasting tool developed by Facebook in 2017 [27,28]. It is capable of handling time series data exhibiting both linear and nonlinear characteristics, as well as effectively capturing seasonal effects. Owing to its simplicity, computational efficiency, and ability to automatically fit future trends, the Prophet model has been widely used; it can autonomously identify underlying patterns and evolutionary trends within the data, thereby generating highly accurate predictions [29]. The Ljung-Box *Q* test was used to examine residual autocorrelation in the Prophet model. Its basic mathematical formulation is expressed as follows:

*y_t_* = *g*(*t*) + *s*(*t*) + *h*(*t*) + ε*_t_*

In this equation, *g*(*t*) denotes the trend component, reflecting the nonperiodic long-term changes in the time series; *s*(*t*) represents the seasonal component, characterizing the periodic fluctuations of the time series; *h*(*t*) stands for the holiday effect component, evaluating the impact of specific holidays or events on the time series; and ε*_t_* is the error term, representing the white noise sequence that remains unexplained by the model.

#### Holt-Winters Model

The Holt-Winters model, also known as triple exponential smoothing, centers on the core concept of using exponential smoothing techniques to simultaneously estimate the level, trend, and seasonal components of a time series. By dynamically updating these components, the model adaptively captures changing data characteristics and enables multi–step ahead forecasting. The Holt-Winters model is primarily categorized into 2 types: the additive model and the multiplicative model. The former is suitable for scenarios where seasonal fluctuations remain constant regardless of the series level, whereas the latter is appropriate when seasonal fluctuations vary proportionally with the series level. In the field of infectious disease forecasting, the multiplicative model is more commonly used as fluctuations in case numbers are typically highly correlated with the overall scale of the epidemic. The model incorporates 3 smoothing parameters, α, β, and γ, which adjust the weights of the level, trend, and seasonal components, respectively, with each parameter ranging from 0 to 1. The selection of the optimal model is based on the principle of minimizing evaluation metrics such as the Bayesian information criterion, and the model residuals must pass the Ljung-Box *Q* white noise test [30].

#### XGBoost Model

XGBoost is an ensemble learning algorithm based on the gradient boosting framework, widely applied to classification and regression problems [31]. Using decision trees as base learners, the algorithm iterates through an additive model where each newly added decision tree aims to fit the residuals of the model from the previous round. Its core idea is to assemble numerous weak learners with simple structures into a high-precision strong predictive model. This study used a grid search strategy to optimize the hyperparameters of the XGBoost model. The parameter space to be optimized included the number of iterations (nrounds), maximum tree depth (max_depth), feature sampling rate (colsample_bytree), minimum sum of instance weight in a leaf (min_child_weight), subsampling rate (subsample), learning rate (eta), and the minimum loss reduction required for a node split (gamma). Simultaneously, 5-fold cross-validation was combined to comprehensively evaluate the performance of each parameter combination, ensuring objective evaluation while effectively preventing the model from overfitting to specific datasets [32]. Residual autocorrelation of the XGBoost model was assessed using the Ljung-Box *Q* test. The mathematical expression of the model is as follows:

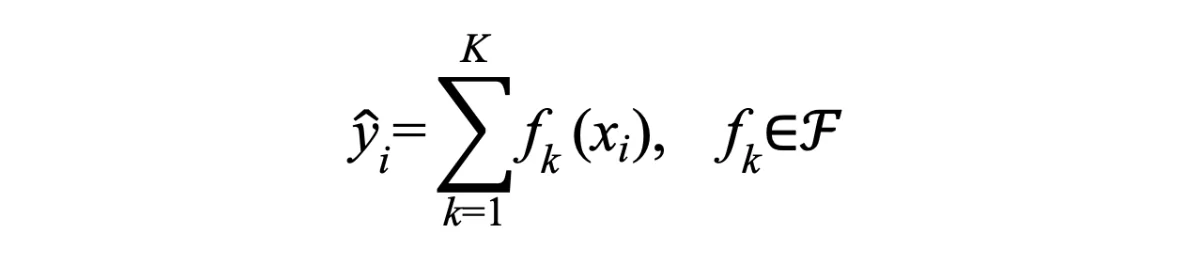

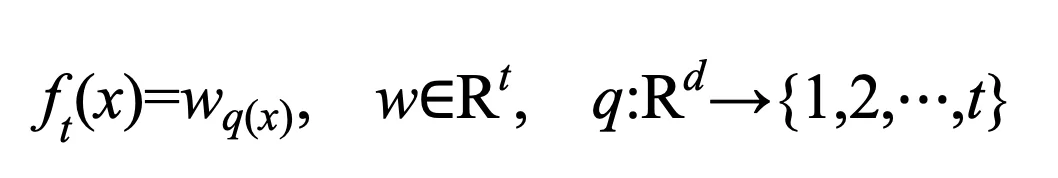





In this equation, *ŷ_i_* represents the predicted value of the sample, *x_i_* represents the input feature vector, *f_k_*(*x_i_*) represents the output score of the *k*th decision tree, *K* is the total number of decision trees, and *F* is the function space of all possible tree structures. *q*(*x*) is the tree structure function that maps input features to a specific leaf node, *t* is the total number of leaf nodes in a single tree, and *w_q_*_(_*_x_*_)_ represents the weight of the corresponding leaf node.

#### Evaluation and Comparison of Models

In constructing the aforementioned models, HFRS incidence data from January 2010 to December 2023 were used as the training set, and data from January 2024 to December 2024 were used as the testing set. This study used 3 metrics, specifically, root mean squared error (RMSE), mean absolute error (MAE), and mean absolute percentage error (MAPE), to evaluate the predictive performance of the models. Smaller values of these metrics indicate higher forecasting accuracy of the models. The calculation formulas for each metric are as follows:

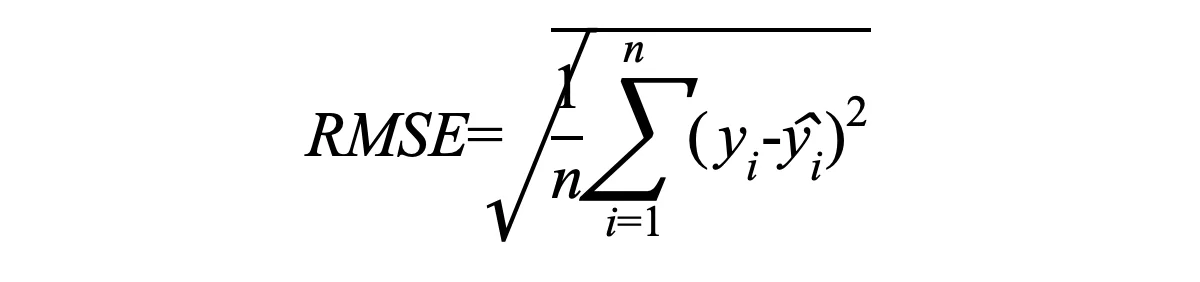

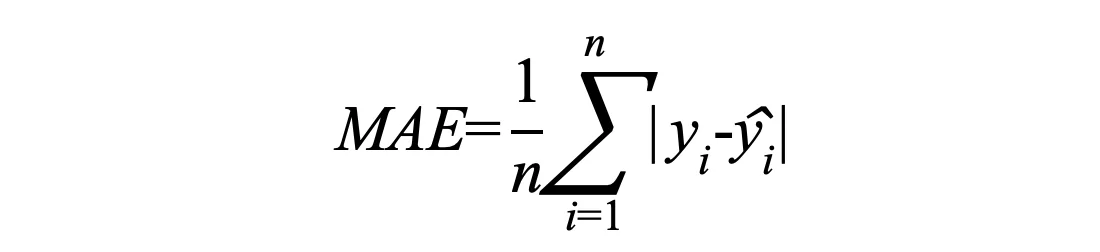

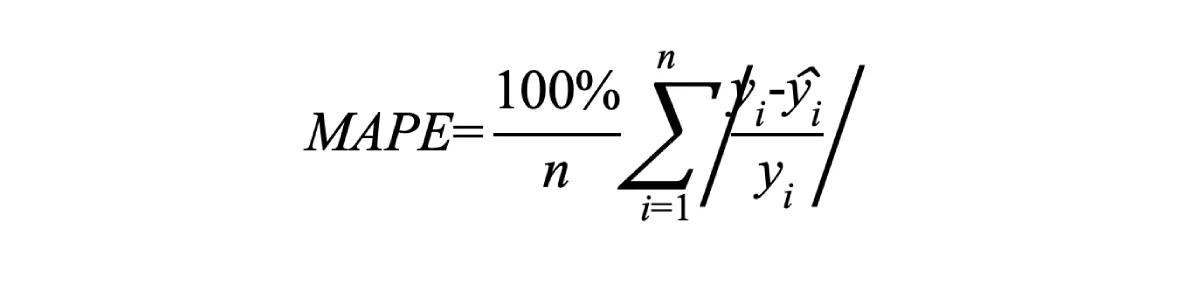







In these equations, *n* is the number of samples, *y_i_* is the actual value of the *i*th sample, and *ŷ_i_* is the predicted value of the *i*th sample.

### Ethical Considerations

This study was approved by the ethics committee of the Shandong Provincial Center for Disease Control and Prevention (SDJK2026-067-01). Given that this study analyzed anonymized data sourced from the China Information System for Disease Control and Prevention, the ethics committee waived the requirement for additional informed consent. All participant-related information was processed under strict confidentiality safeguards, and no compensation was provided to participants in this study.

## Results

### Epidemiological Characteristics

#### Temporal Characteristics

From 2010 to 2024, a total of 15,184 HFRS cases were reported in Shandong Province, with the annual reported incidence rate ranging from 0.45 to 1.84 per 100,000 population. Overall, the incidence rate of HFRS showed a trend of an initial increase followed by a decrease, peaking in 2013. During the period from 2013 to 2024, the number of HFRS cases exhibited a fluctuating downward trend, reaching its lowest level in 2024. Of the 15,184 cases, a total of 188 HFRS deaths were reported during the study period. The disease was reported in all months of the year, demonstrating substantially seasonal distribution characteristics; the incidence peak was concentrated between October and December, accounting for 52.8% (8011 cases) of the total cases (Figures 2 and 3).

**Figure 2:**
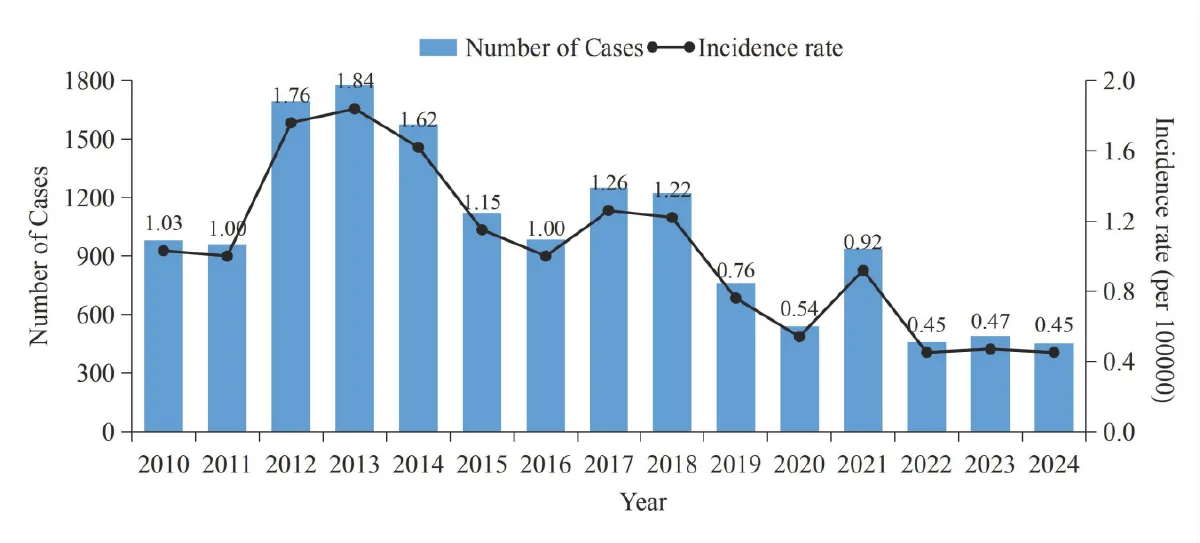
General overview of hemorrhagic fever with renal syndrome incidence in Shandong Province from 2010 to 2024.

**Figure 3:**
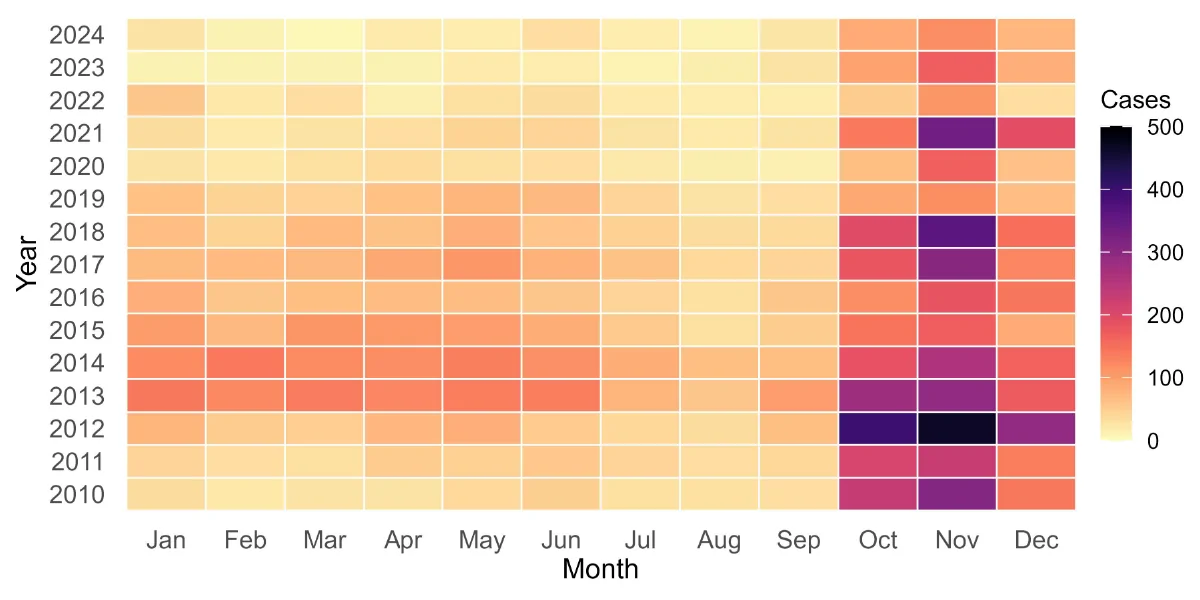
Heat map of monthly hemorrhagic fever with renal syndrome incidence in Shandong Province from 2010 to 2024.

#### Spatial Characteristics

From 2010 to 2024, all 16 prefecture-level cities in Shandong Province reported cases of HFRS. Of the 136 counties (cities and districts) in the province, 135 reported cases, with the sole exception of Pingyuan County in the city of Dezhou. The top 5 counties (cities and districts) in terms of average annual incidence rates were Wulian County (6.08 per 100,000), the city of Haiyang (5.25 per 100,000), Yiyuan County (4.77 per 100,000), the city of Zhucheng (3.77 per 100,000), and Donggang District (3.59 per 100,000), which are mainly concentrated in the eastern and southeastern regions of Shandong Province. Regarding the evolution of the annual spatial distribution, the geographical extent affected by the HFRS epidemic exhibited a gradually shrinking trend, and the number of high-incidence counties (cities and districts) also decreased year by year (Figure 4).

**Figure 4:**
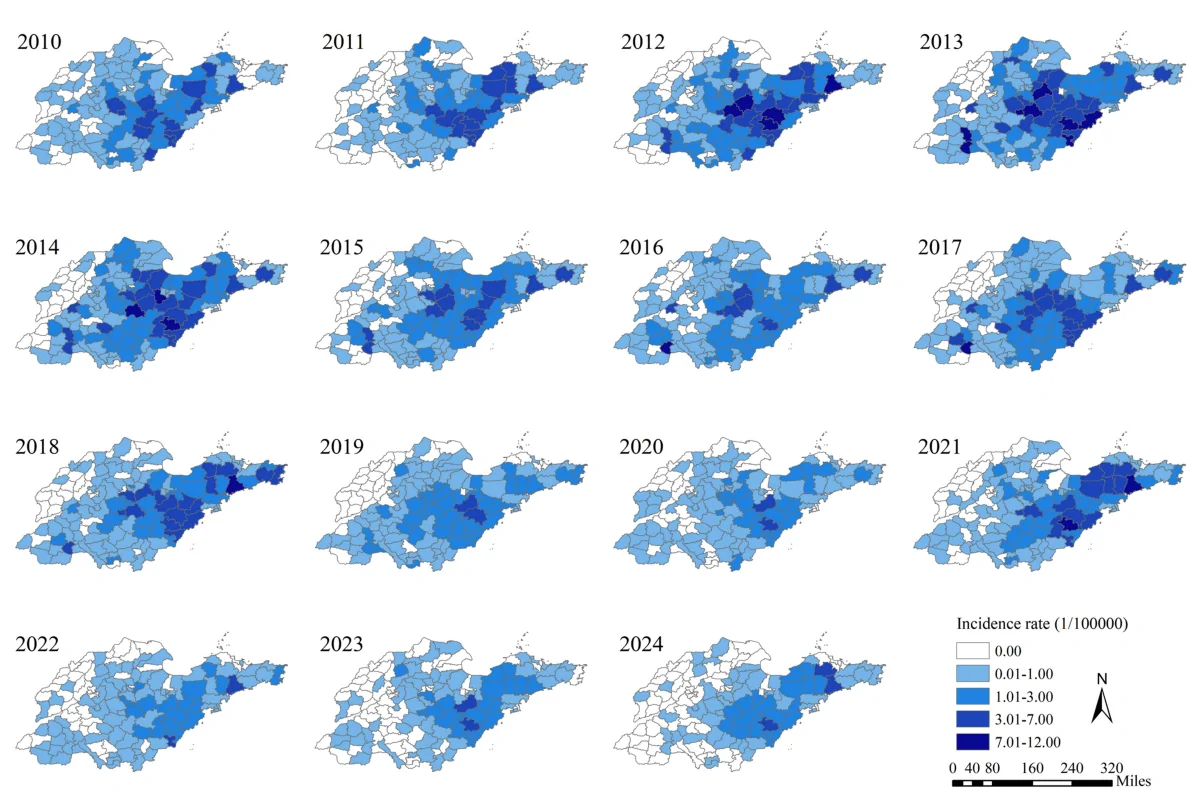
Distribution of hemorrhagic fever with renal syndrome incidence in Shandong Province from 2010 to 2024.

#### Demographic Characteristics

Among the total HFRS cases reported from 2010 to 2024, there were 11,023 (N=15,184) male individuals and 4161 female individuals, with a male-to-female ratio of 2.65:1. The mean age was 49.91 (SD 14.96) years, and patients were predominantly middle-aged and older adults aged 40 to 69 years, totaling 10,372 (N=15,184) cases. The top 3 occupations were farmers (12,609 cases), workers (896 cases), and houseworkers or unemployed individuals (458 cases; Table 1).

**Table 1:** Demographic characteristics of hemorrhagic fever with renal syndrome cases in Shandong Province from 2010 to 2024 (N=15,184).

Categories		Cases, n (%)	Deaths (n=188), n (%)
**Sex**			
	Male	11,023 (72.6)	140 (74.5)
	Female	4161 (27.4)	48 (25.5)
**Age (years)**			
	0-9	60 (0.4)	0 (0)
	10-19	392 (2.6)	1 (0.5)
	20-29	1098 (7.2)	4 (2.1)
	30-39	1952 (12.9)	9 (4.8)
	40-49	3559 (23.4)	40 (21.3)
	50-59	4086 (26.9)	59 (31.4)
	60-69	2727 (18.0)	53 (28.2)
	70-79	1047 (6.9)	14 (7.4)
	≥80	263 (1.7)	8 (4.3)
**Occupation**			
	Student	343 (2.3)	1 (0.5)
	Teacher	60 (0.4)	0 (0)
	Business service	81 (0.5)	1 (0.5)
	Medical personnel	29 (0.2)	0 (0)
	Worker	896 (5.9)	9 (4.8)
	Farmer	12,609 (83.0)	155 (82.4)
	Clerical staff	165 (1.1)	3 (1.6)
	Retired personnel	260 (1.7)	6 (3.2)
	Houseworker or unemployed	458 (3.0)	9 (4.8)
	Unknown	68 (0.4)	1 (0.5)
	Other	215 (1.4)	3 (1.6)

### Time Series Analysis

#### SARIMA Model

The augmented Dickey-Fuller test rejected the unit-root null hypothesis for the HFRS incidence series (*P*=.01). STL decomposition nevertheless revealed a clear seasonal pattern, with a seasonal period of 12 months (Figure 5). On the basis of the ACF and partial ACF plots and subsequent model screening using the *auto.arima* function, SARIMA(1, 1, 1) (0, 1, 1)_12_ was selected as the optimal model, with the lowest Akaike information criterion value of 237.81. The fitting performance of the optimal model is shown in Figure 6. The Ljung-Box *Q* test showed no significant residual autocorrelation (*P*=.80; Figure 7). The RMSE, MAE, and MAPE of the model were 11.08, 9.20, and 46.67%, respectively.

**Figure 5:**
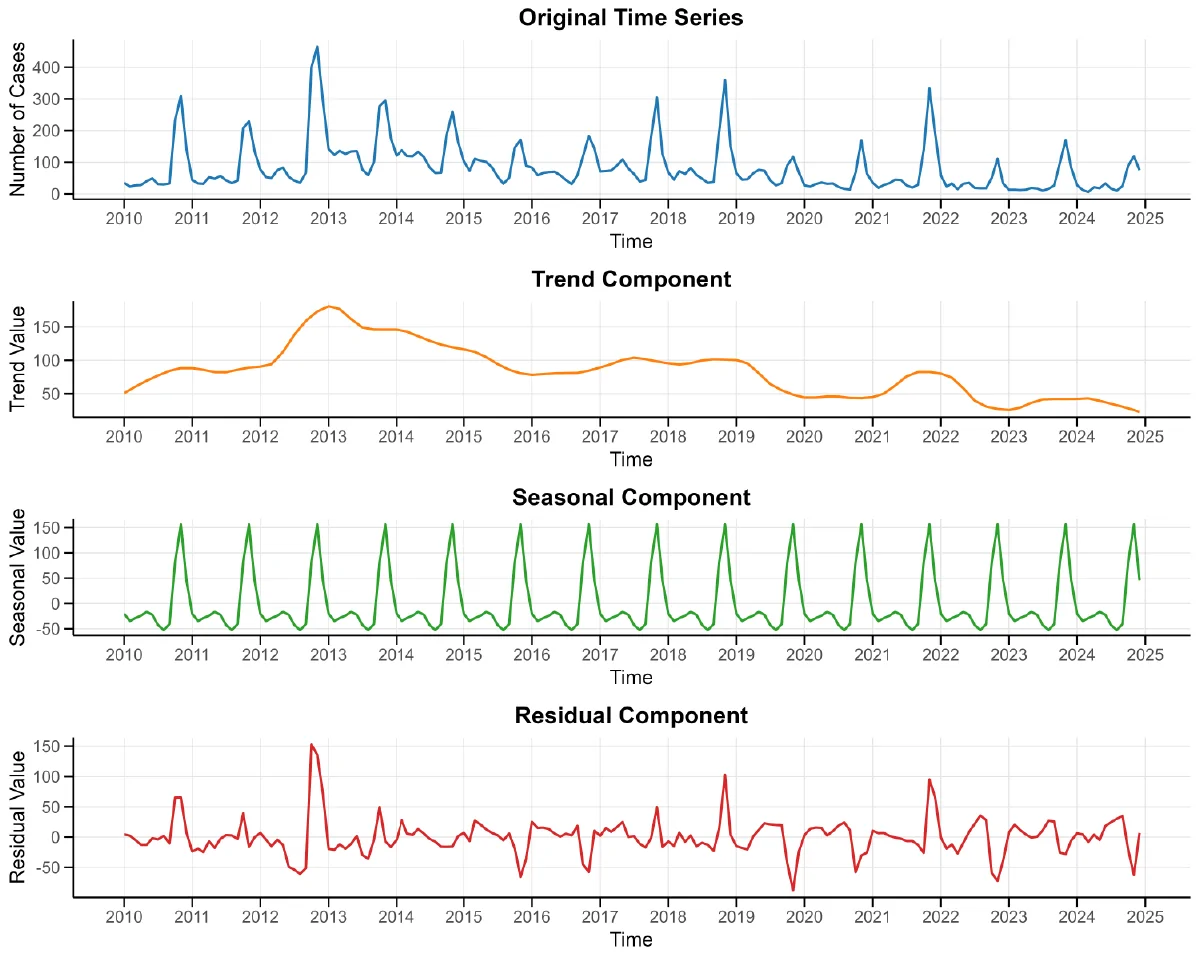
Seasonal-trend decomposition using locally estimated scatterplot smoothing–based seasonal decomposition of hemorrhagic fever with renal syndrome cases in Shandong Province from 2010 to 2024.

**Figure 6:**
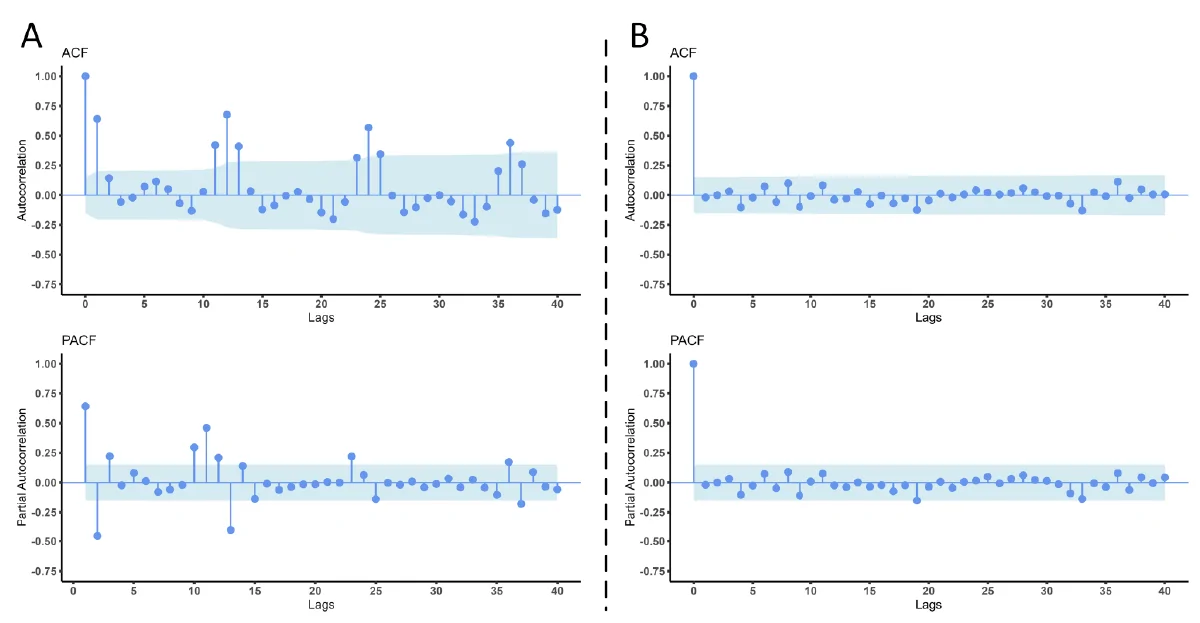
The autocorrelation function (ACF) and partial ACF (PACF) plots of monthly incidence of hemorrhagic fever with renal syndrome in Shandong Province from 2010 to 2024: (A) the original time series and (B) the adjusted time series.

**Figure 7:**
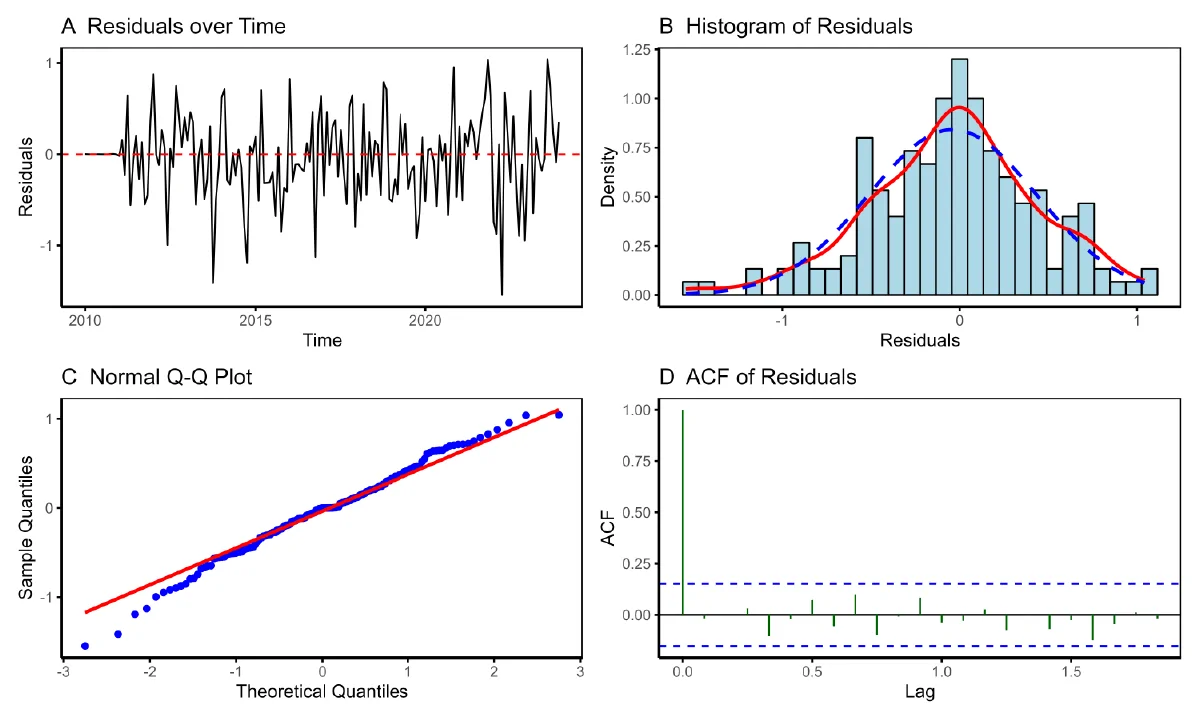
Residual analysis of the seasonal autoregressive integrated moving average(1, 1, 1) (0, 1, 1)12 model: (A) residuals over time, (B) histogram of residuals, (C) normal Q-Q plot of residuals, and (D) autocorrelation function (ACF) plot of residuals.

#### Prophet Model

In the construction of the Prophet model, given the declining trend and annual seasonality of HFRS incidence in Shandong Province, the trend growth parameter was set to linear, the yearly seasonality parameter was set to TRUE, the seasonality mode was set to multiplicative, the seasonality prior scale was set to 10, and the change point prior scale was set to 0.05 (Table 2). Significant residual autocorrelation was detected for the Prophet model through the Ljung-Box *Q* test (*P*<.001). The RMSE, MAE, and MAPE of the model were 11.28, 9.45, and 68.08%, respectively.

**Table 2:** Parameter values of the constructed Prophet, Holt-Winters, and Extreme Gradient Boosting (XGBoost) models.

Models and parameters			Values
**Prophet**			
	Growth	Linear	
	Yearly seasonality	TRUE	
	Seasonality mode	Multiplicative	
	Seasonality prior scale	10	
	Change point prior scale	0.05	
**Holt-Winters**			
	α	0.18	
	β	0.12	
	γ	0.05	
	Seasonal	Multiplicative	
**XGBoost**			
	Number of iterations	1500	
	Maximum tree depth	6	
	Learning rate	0.05	
	Minimum loss reduction required for a node split	0.1	
	Feature sampling rate	1	
	Minimum sum of instance weight in a leaf	3	
	Subsampling rate	0.6	

#### Holt-Winters Model

The results of the Holt-Winters model analysis showed that, through automatic screening in the R software, the multiplicative model was ultimately identified as the optimal fitting model. The smoothing parameters α, β, and γ of the model were 0.18, 0.12, and 0.05, respectively (Table 2). The residual series test indicated that the model residuals exhibited white noise characteristics (*P*=.18). The RMSE, MAE, and MAPE of the model were 7.56, 6.43, and 34.56%, respectively.

#### XGBoost Model

The results of the XGBoost model analysis showed that, through grid search and 5-fold cross-validation optimization, the optimal parameter combination for the model was determined as follows: 1500 iterations (nrounds), maximum depth (max_depth) of 6, column sampling rate (colsample_bytree) of 1, minimum child weight (min_child_weight) of 3, subsampling rate (subsample) of 0.6, learning rate (eta) of 0.05, and gamma coefficient (gamma) of 0.1 (Table 2). The residuals of the XGBoost model showed significant autocorrelation (Ljung-Box *Q* test; *P*<.001). The RMSE, MAE, and MAPE of the model were 16.94, 10.96, and 55.66%, respectively.

#### Model Comparison

Three evaluation metrics (RMSE, MAE, and MAPE) were used to assess the predictive performance of each model. In the 2024 test set, the Holt-Winters model yielded the lowest values for all 3 metrics among the 4 models. On the basis of these point estimate error metrics, SARIMA, Prophet, and XGBoost showed progressively higher prediction errors (Figure 8; Table 3).

**Figure 8:**
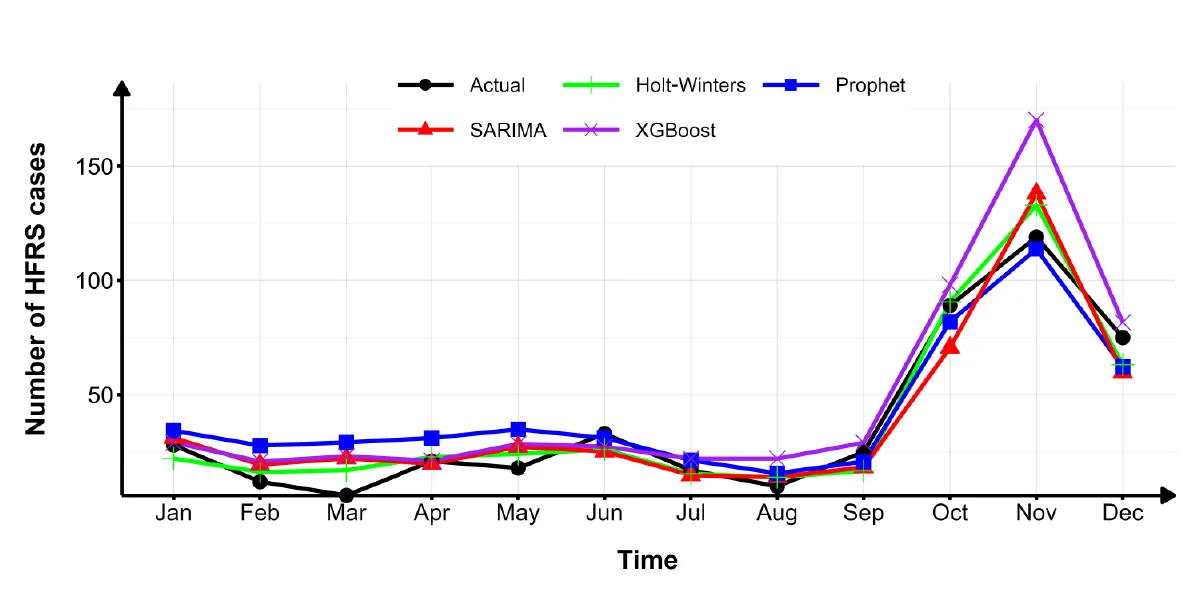
Comparison of the actual hemorrhagic fever with renal syndrome (HFRS) cases with the predicted cases by the 4 models from January 2024 to December 2024. SARIMA: seasonal autoregressive integrated moving average; XGBoost: Extreme Gradient Boosting.

**Table 3:** Comparison of the 4 models using root mean squared error (RMSE), mean absolute error (MAE), and mean absolute percentage error (MAPE).

	SARIMA^a^	Prophet	Holt‑Winters	XGBoost^b^
RMSE	11.08	11.28	7.56	16.94
MAE	9.20	9.45	6.43	10.96
MAPE (%)	46.67	68.08	34.56	55.66

^a^SARIMA: seasonal autoregressive integrated moving average.

^b^XGBoost: Extreme Gradient Boosting.

## Discussion

As a major endemic region for HFRS in China, Shandong Province has experienced an overall decline in incidence in recent years. However, HFRS’s seasonality, spatial clustering, and high-risk population characteristics remain significant, indicating that the underlying endemicity has not fundamentally changed [17]. To further translate epidemiological research into practical prevention, control, and early warning capabilities, this study systematically analyzed 15 years of HFRS surveillance data in Shandong Province and comparatively evaluated 4 prediction models. The results indicate significant spatiotemporal heterogeneity in the transmission of HFRS in Shandong Province, with the Holt-Winters model demonstrating superior short-term predictive performance. These findings contribute to a deeper understanding of the epidemiological characteristics of HFRS and provide a methodological reference for implementing routine surveillance and early warning. Unlike our previous studies [16,17], which mainly focused on spatiotemporal patterns and driving factors of HFRS, the present study examined the long-term epidemiological trends of HFRS in Shandong Province based on a 15-year time series dataset (2010-2024). This extended observation window enabled a more systematic profiling of temporal dynamics, spatial heterogeneity, and demographic changes, together with the evaluation of multiple time series forecasting models. The present study addressed a distinct research question and provided complementary, nonredundant rather than repetitive evidence.

In terms of long-term epidemic trends, this study found that the incidence of HFRS in Shandong Province exhibited a fluctuating downward trend, consistent with the overall epidemic trend in China in recent years [33,34]. Previous studies have indicated that although China still bears the heaviest global burden of HFRS, the national incidence has gradually decreased over the past decades. This decline may be attributed to multiple factors, including the promotion of vaccination, implementation of rodent control measures, improvement of living conditions, and strengthening of disease surveillance [35]. However, the decline in HFRS incidence does not imply the disappearance of its transmission risk as Shandong still possesses the ecological foundation to sustain endemicity in terms of geographical environment, host animal distribution, and population exposure patterns [14]. Furthermore, this study revealed that reported HFRS cases were primarily clustered between October and December, which differs from the previously bimodal seasonal distribution reported in Shandong Province [17]. This finding indicates a potential shift in the recent seasonal pattern of HFRS toward autumn-winter predominance. Previous studies have shown that HFRS seasonality may be influenced by changes in rodent host population composition, circulating hantavirus types, and opportunities for human exposure [36]. Typically, HTNV-related HFRS cases peak in autumn and winter, whereas SEOV-associated cases tend to show a spring peak [1,37]. Accordingly, the diminished spring incidence peak observed in our study may be attributable to altered predominance of hantavirus types, the spatial distribution of rodent hosts, and seasonal patterns of human-rodent contact. However, the precise mechanisms remain to be explored through further virological and ecological investigations for validation.

HFRS has long been influenced by ecological environments and socioeconomic conditions, which leads to disparities in its regional distribution [8,38]. This study revealed that high-incidence counties for HFRS were primarily located in eastern and southeastern Shandong, whereas both the geographic extent of the epidemic and the number of high-incidence counties gradually decreased in recent years. Previous studies have shown that HFRS occurrence is associated with altitude, land use, and population density, suggesting that ecological suitability for rodent hosts and opportunities for human exposure may jointly influence infection risk [14]. Additional ecological studies in Shandong further showed that land cover and elevation were closely associated with the distribution of hantavirus-infected rodent hosts, whereas southeastern Shandong has been identified as an important natural focus characterized by the presence of striped field mouse and HTNV [39]. Agricultural field activities may further increase opportunities for contact with infected rodents and their contaminated environments [40]. Such ecological and occupational factors may partly contribute to the relatively high incidence observed in eastern and southeastern Shandong. Previous studies have also demonstrated substantial spatial heterogeneity in HFRS across Shandong Province, indicating that provincial-level unified prevention and control measures are insufficient; geographically tailored and risk-stratified intervention strategies should therefore be prioritized [16]. Additionally, consistent with previous findings, HFRS cases were predominantly among middle-aged and older adult male individuals, with farmers representing the highest proportion in occupational distribution [24,41]. The differential distribution of HFRS among the population is likely driven by variations in exposure opportunities. Male farmers often engage in outdoor cultivation, leading to more frequent contact with rodents or their excreta, which suggests that agricultural production settings remain the core focus for HFRS prevention and control [42]. Furthermore, the outflow of young rural labor, the aging of the agricultural workforce, and the fact that HFRS vaccines are not administered to individuals over 60 years of age may also contribute to the high proportion of middle-aged and older adult individuals among HFRS cases [43]. This suggests that middle-aged and older adult male individuals in rural areas should be the primary targets for health education, advocating for active HFRS vaccination and the use of personal protection during labor.

This study shows that all 4 models can effectively capture the seasonal fluctuations of HFRS; however, the Holt-Winters model yielded the lowest RMSE, MAE, and MAPE, outperforming the SARIMA, Prophet, and XGBoost models. This result suggests that for monthly case sequences with a clear annual cycle, a relatively stable long-term trend, and a limited training sample size, structurally simple exponential smoothing methods still possess significant practical value. By separately smoothing and updating the level, trend, and seasonal components, the Holt-Winters model can stably track monthly fluctuations without introducing excessive parameters, making it particularly suitable for data dominated by significant seasonality rather than abrupt changes [30,44]. The overall performance of the SARIMA model was slightly inferior to that of the Holt-Winters model, yet it remains one of the most widely used models in previous HFRS time series analyses. For instance, research in Jiaonan County, Shandong Province, has shown that SARIMA could accurately predict local monthly HFRS incidence, and national-scale studies have also demonstrated its ability to effectively simulate long-term seasonal fluctuations of HFRS [45,46]. The disadvantage of SARIMA lies in its reliance on stationarity and linearity assumptions, and its parameter identification is highly dependent on the sequence structure; thus, its extrapolation capability may be limited when the epidemic is influenced by policy changes or abnormal exposure events [23]. The Prophet model is suitable for time series with prominent trend shifts or holiday disturbances [47]. However, as the changes in monthly HFRS cases primarily reflect fixed seasonality and local volatility, the advantages of the Prophet model were not fully realized in this study. The XGBoost model performed the worst in this study, which contradicts the “naturally superior” expectation some scholars hold for machine learning models. The advantages of machine learning methods are mainly reflected in handling complex nonlinear relationships and high-dimensional features, but their performance typically depends on large training sample sizes, sufficient feature engineering, and the inclusion of external variables. This study modeled HFRS using only monthly case data without incorporating features such as meteorology, host density, or land use that could improve nonlinear fitting capabilities; therefore, the XGBoost model could not fully leverage its strengths. In addition, the residuals of the Prophet and XGBoost models exhibited significant autocorrelation, indicating that neither model fully captured the temporal dependencies in HFRS incidence. This may be attributable to the complex temporal dynamics of HFRS, including evolving seasonal patterns, interannual fluctuations, and occasional abrupt changes in incidence. Therefore, although these models were able to fit the overall temporal trends reasonably well, they remained somewhat limited in characterizing fine-scale temporal variations.

This study also has certain limitations. First, the HFRS case data were obtained from a passive surveillance information system, which may involve reporting biases such as underreporting and diagnostic delays. Second, although the 2024 test set covered a complete annual seasonal cycle, its relatively limited sample size may have affected the robustness of the model comparisons to some extent. Future studies should consider using data spanning longer periods for validation to further confirm the reliability of the models. Finally, to ensure consistency in the input variables across the 4 forecasting models and assess their applicability under routine surveillance conditions, the XGBoost model in this study incorporated only historical monthly HFRS case counts. However, this univariate design may constrain the advantage of XGBoost in capturing multivariable nonlinear relationships. Future studies should further explore incorporating meteorological conditions, rodent dynamics, ecological and environmental factors, and socioeconomic indicators into multivariable models to further improve prediction performance.

In conclusion, the HFRS epidemic in Shandong Province features low overall incidence at the provincial level, whereas specific local foci continue to exhibit high incidence. The incidence of HFRS shows significant spatiotemporal clustering, including distinct autumn and winter epidemic peaks, obvious geographical concentrations in eastern and southeastern Shandong, and high-risk groups dominated by middle-aged and older adult male farmers. This suggests that future prevention and control efforts should focus more on spatially precise strategies and targeted interventions for high-risk populations. For short-term forecasting based on monthly surveillance data, the Holt-Winters model yielded the lowest prediction error metrics among the evaluated models and may be a useful practical tool for routine provincial HFRS early warning. The results of this study have important implications for the precise monitoring and scientific prevention and control of regional HFRS.

## Data Availability

The datasets used and/or analyzed during the current study are available from the corresponding author on reasonable request.

## Abbreviations

ACF: autocorrelation function
HFRS: hemorrhagic fever with renal syndrome
HTNV: Hantaan virus
MAE: mean absolute error
MAPE: mean absolute percentage error
RMSE: root mean squared error
SARIMA: seasonal autoregressive integrated moving average
SEOV: Seoul virus
STL: seasonal-trend decomposition using locally estimated scatterplot smoothing
XGBoost: Extreme Gradient Boosting
